# Fecal Calprotectin Predicts Mucosal Healing in Patients With Ulcerative Colitis Treated With Biological Therapies: A Prospective Study

**DOI:** 10.14309/ctg.0000000000000174

**Published:** 2020-05-18

**Authors:** Lorenzo Bertani, Corrado Blandizzi, Maria Gloria Mumolo, Linda Ceccarelli, Eleonora Albano, Gherardo Tapete, Giovanni Baiano Svizzero, Federico Zanzi, Francesca Coppini, Nicola de Bortoli, Massimo Bellini, Riccardo Morganti, Santino Marchi, Francesco Costa

**Affiliations:** 1 Department of Translational Research and New Tecnologies in Medicine and Surgery, University of Pisa, Pisa, Italy;; 2 Department of Clinical and Experimental Medicine, University of Pisa, Pisa, Italy;; 3 IBD Unit, Department of General Surgery and Gastroenterology, Pisa University Hospital, Pisa, Italy;; 4 Section of Statistics, Pisa University Hospital, Pisa, Italy.

## Abstract

Biological therapies are widely used for the treatment of ulcerative colitis. However, only a low proportion of patients achieve clinical remission and even less mucosal healing. There is currently scarce knowledge about the early markers of therapeutic response, with particular regard to mucosal healing. The aim of this prospective study was to evaluate the role of fecal calprotectin (FC) as early predictor of mucosal healing.

**METHODS:**

A prospective observational study was conducted on patients with ulcerative colitis, who started biological therapy with infliximab, adalimumab, golimumab, or vedolizumab at our center. All patients underwent colonoscopy, performed by 2 blinded operators, at baseline and week 54 or in case of therapy discontinuation because of loss of response. FC was assessed at baseline and week 8 and evaluated as putative predictor of mucosal healing at week 54.

**RESULTS:**

We enrolled 109 patients, and 97 were included in the analysis. Twenty-six patients (27%) experienced loss of response. Over 71 patients (73%) with clinical response at week 54, clinical remission was obtained in 60 patients (61.9%) and mucosal healing in 45 patients (46.4%). After 8 weeks of treatment, FC predicted mucosal healing at week 54 (*P* < 0.0001). Sensitivity, specificity, positive predictive value, and negative predictive value were estimated to be 75%, 88.9%, 86.6%, and 75.5%, respectively, based on a cutoff of 157.5 mg/kg.

**DISCUSSION:**

The present study suggests that FC assessment after 8 weeks of treatment with all the biological drugs could represent a promising early marker of response to therapy in terms of mucosal healing.

## INTRODUCTION

Ulcerative colitis (UC) is a chronic relapsing disease that involves the colorectal mucosa. Over the years, the therapeutic target has been upgraded from the resolution of symptoms to deep remission to prevent relapses and complications. With this goal, the STRIDE consensus suggested that the primary therapeutic target to be achieved in patients with UC is both clinical/symptomatic (defined as resolution of rectal bleeding and diarrhea/altered bowel habit) and endoscopic remission (1). In this respect, mucosal healing is regarded as an indispensable treatment outcome because it serves as a validated surrogate marker for the effective control of the disease and, thereby, its positive course over the time (2,3). There is no complete agreement in defining mucosal healing, but an international consensus defined it as the absence of friability, blood, erosions, and ulcers of the bowel mucosa (4) corresponding to a Mayo Endoscopic Score (MES) of 0 or 1.

The anti–tumor necrosis factor (TNF) monoclonal antibodies infliximab (IFX), adalimumab (ADA), and golimumab (GOL) have greatly improved treatment expectations in patients with UC refractory or intolerant to standard treatments (5,6), allowing to achieve and maintain clinical remission and mucosal healing (7–9). However, a substantial proportion of patients experience primary nonresponse or loss of response to anti-TNF treatment. To overcome this issue, a biodrug with a different mechanism of action, vedolizumab (VDZ), has been developed. VDZ is a monoclonal antibody that targets α_4_β_7_ integrin expressed in a subset of T lymphocytes, preventing their endothelial adherence and migration toward the bowel mucosa (10). It is able to induce clinical remission and mucosal healing, even in patients who experienced loss of response to anti-TNF drugs (11–13). However, even a non-negligible number of patients have been found to develop loss of response during VDZ treatment (14,15).

The early prediction of response to biological therapies is one of the most important challenges for the clinicians. In this respect, the identification of a reliable biomarker would allow to optimize the management of patients with UC, improving the cost-effectiveness of biological therapies (16).

Calprotectin is a 36-kDa calcium- and zinc-binding protein, which represents approximately 60% of soluble proteins of granulocyte cytoplasm (17). Fecal calprotectin (FC) is strongly correlated with both MES and Ulcerative Colitis Endoscopic Score (18,19). In previous studies, FC was shown to be helpful in predicting sustained clinical remission (20) and mucosal healing (21,22) during anti-TNF treatment, particularly with IFX and ADA. However, no investigations have been performed to evaluate the predictive value of FC in terms of mucosal healing in a prospective cohort of patients with UC treated with IFX, ADA, GOL, and VDZ. Based on the above background, the aim of the present prospective study is to identify a reliable biomarker able to predict therapeutic effectiveness in UC, regardless of the type of biodrug, which could significantly improve therapeutic management.

## MATERIALS AND METHODS

### Patients and study design

Consecutive adult patients with UC, who underwent biological treatment with IFX (biosimilar CT-P13), ADA, GOL, or VDZ at the Pisa University Hospital from October 2016 to March 2018, were enrolled prospectively. Patients had to meet the indications for treatment with TNF antagonists or VDZ and agreed to participate in the study signing informed consent. Patients enrolled in this prospective study the day of the first dose of the biological treatment and were evaluated every 8 weeks for 54 weeks. We excluded from the analysis patients who were primary nonresponders to anti-TNF or VDZ therapy, defined as a decrease in Full Mayo Score ≤2 or a lack of improvement of rectal bleeding at week 8. Patients treated concomitantly with immunosuppressants were excluded as well. Treatment regimen was as follows:

1. 5 mg/kg i.v. at weeks 0, 2, and 6 and then every 8 weeks for IFX;
2. 160 mg at week 0, 80 mg at week 2, and 40 mg subcutaneously starting from week 4 for ADA;
3. 200 mg at week 0, 100 mg at week 2, and then 50 mg or 100 mg (if patient's weight was >80 kg) subcutaneously every 4 weeks for GOL; and
4. 300 mg at weeks 0, 2, and 6 and then every 8 weeks for VDZ.

Treatments with IFX and VDZ could be escalated after week 6 to a monthly regimen, whereas ADA treatment could be escalated to a weekly regimen, in case of a mild worsening of UC after a careful medical evaluation, which included testing for antidrug antibodies. We recorded general patient features, such as age, sex, age at diagnosis, previous immunosuppressant, and previous anti-TNF treatment; moreover, baseline disease extension, concomitant corticosteroid treatment, FC, Partial Mayo Score (PMS), and MES were recorded.

A team of clinicians performed clinical evaluations, defining clinical status by PMS. At each time point, FC and C-reactive protein (CRP) were assessed. FC was determined using the ELISA Bühlmann fCAL Turbo (Bühlmann Laboratories AG, Schönenbuch, Switzerland), known to perform with high sensitivity and specificity (23).

Colonoscopy was performed at baseline and after 54 weeks of treatment to evaluate the therapeutic outcome in terms of mucosal healing. In case of loss of response (after at least 14 weeks of treatment) with a subsequent discontinuation of therapy, the therapeutic outcome was assessed on the basis of endoscopy and clinical examination at discontinuation. All colonoscopies were performed by 2 operators expert in the evaluation of MES, blinded to the results of FC and CRP. Mucosal healing was obtained in case of MES ≤1. Clinical remission was defined with a PMS ≤1. A value of CRP <0.5 mg/dL was considered normal.

### Statistical analysis

The primary endpoint was to evaluate whether FC after 8 weeks of treatment could be used as a possible predictive marker of mucosal healing or clinical remission after 54 weeks of treatment in patients with UC treated with anti-TNF or VDZ in monotherapy. The secondary endpoint was to evaluate the role of CRP after 8 weeks as a possible marker of mucosal healing or clinical remission after 54 weeks of treatment.

To evaluate the primary endpoint, we set power, α error, and effect size at 95%, 5%, and 60 mg/kg as delta FC between the responders and nonresponders group in terms of mucosal healing, respectively. At least 40 patients per group were needed in both the groups. According to real-life studies, we expected a rate of primary nonresponders of 20% (24,25) and a rate of mucosal healing of 40%–50% (26,27); therefore, we planned to enroll 100–110 patients.

Categorical data were described by frequency (%) and quantitative data by mean (SD) or median (interquartile range). To compare FC and CRP with mucosal healing and clinical remission, the Mann–Whitney test (2 tailed) was applied. The receiver operating characteristic (ROC) curve analysis (using a nonparametric test) was performed to calculate the best cutoff in predicting either mucosal healing or clinical remission. Sensitivity, specificity, positive predictive value, and negative predictive value were also calculated. A comparison between drugs in terms of FC levels was performed by the Kruskal–Wallis test. Significance was fixed at 0.05. All analyses were performed using SPSS technology (v.25).

### Ethical considerations

This study was conducted in full compliance with the 1975 Declaration of Helsinki and was approved by the Ethical Committee of the Pisa University Hospital (CEAVNO). All of the included patients signed informed consent forms, approving the use of their anonymized data for research purposes.

## RESULTS

### Study population

During the enrollment period, 109 patients with UC started a biological treatment. We excluded 9 primary nonresponder patients and 3 patients treated concomitantly with azathioprine, thus including 97 patients in the analysis. Twenty-seven patients were treated with IFX, 20 with ADA, 18 with GOL, and 32 with VDZ. Their general characteristics are displayed in Table 1. Overall, disease severity was moderate to severe. Indeed, median PMS was 6, and all patients had 2 or 3 points of MES at baseline; accordingly, FC levels at baseline presented a median value of 355 mg/kg. There were no significant differences between FC levels at baseline in patients stratified by the 4 drugs (*P* = 0.332).

**Table 1.**
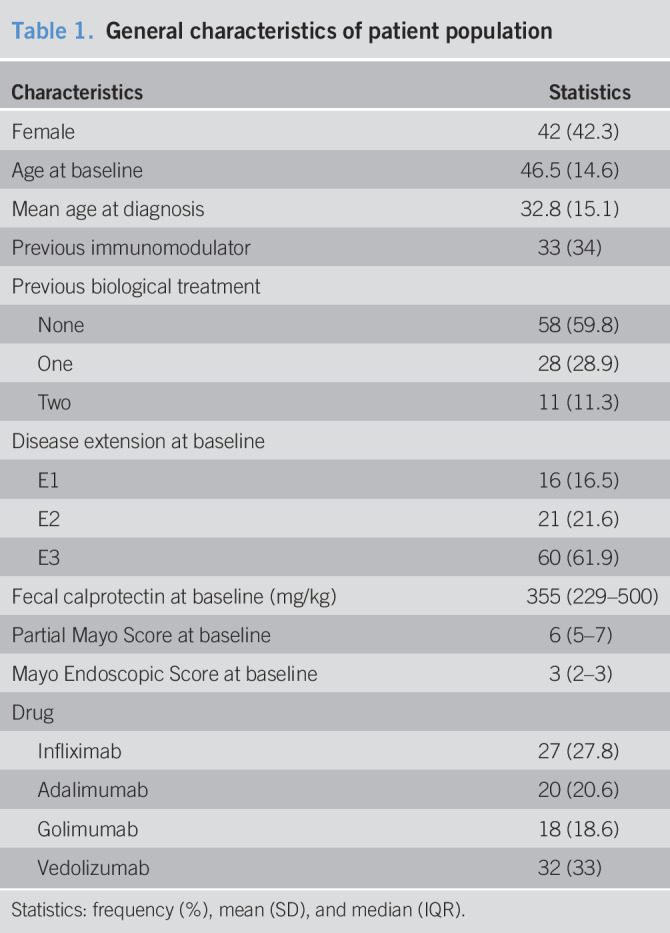
General characteristics of patient population

During the follow-up, 26 patients (27%) experienced loss of response (5 treated with IFX, 6 with ADA, 10 with GOL, and 5 with VDZ): 10 at week 16, 8 at week 24, 3 at week 32, 2 at week 40, and 3 at week 48. Over 71 patients (73%) with clinical response at week 54, clinical remission was achieved in 60 patients (61.9%) and mucosal healing in 45 patients (46.4%). Clinical remission rate was 44.4% in patients treated with IFX, 65% with ADA, 61.1% with GOL, and 75% with VDZ, whereas mucosal healing rates were 29.6%, 60%, 38.9%, and 56.2%, respectively.

### Correlation between FC, C-reactive protein, and clinical and endoscopic outcomes

FC at baseline did not correlate with either mucosal healing or clinical remission at week 54 (*P* = 0.850 and *P* = 0.665, respectively). However, after 8 weeks of treatment, both FC and CRP correlated with clinical remission (*P* < 0.0001 and *P* = 0.023, respectively), whereas only FC correlated with mucosal healing (*P* < 0.0001), as shown in Table 2. The same results were obtained when patients were stratified into 2 groups, according to the different mechanism of action of the drugs (Table 2), suggesting that FC could be used as an early biomarker of mucosal healing regardless of the mechanism of action of the biologic drug.

**Table 2.**
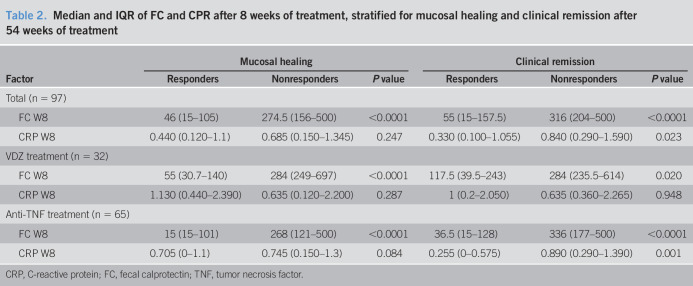
Median and IQR of FC and CPR after 8 weeks of treatment, stratified for mucosal healing and clinical remission after 54 weeks of treatment

**Figure 1.**
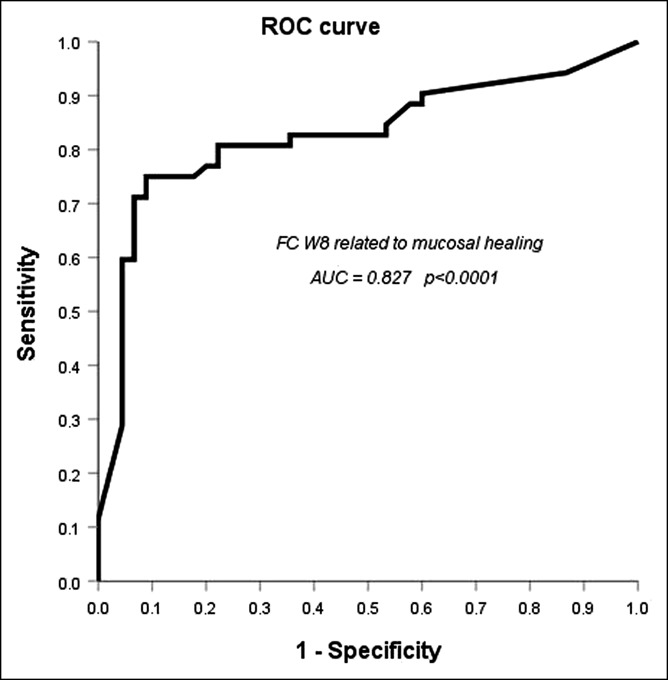
Receiver operating characteristic curve analysis of fecal calprotectin levels at week 8 for the prediction of mucosal healing at week 54. At the cutoff ≤157.5 mg/kg, sensitivity was 75%, specificity 88.9%, positive predictive value 86.6%, and negative predictive value 75.5%.

To calculate the best cutoff of FC at week 8 related to mucosal healing, a ROC analysis was performed (Figure 1), and sensitivity, specificity, positive predictive value, and negative predictive value were calculated: 75%, 88.9%, 86.6%, and 75.5%, respectively, and were obtained choosing a cutoff of 157.5 mg/kg. The ROC analysis of FC values for the prediction of clinical remission is shown in Supplementary Digital Content 1 (see Figure 1, http://links.lww.com/CTG/A285). FC levels were similar in patients treated with the 4 different drugs, without significant differences evaluated by means of the Kruskal–Wallis test (*P* = 0.332 at baseline and *P* = 0.147 at week 8).

## DISCUSSION

This study was conceived to identify an early marker of mucosal healing in a prospective cohort of patients with UC treated with biological therapies, and our results support the view that FC could be regarded as a reliable tool in this perspective. To predict mucosal healing during biological treatment with an accurate, rapid, and noninvasive biomarker is currently one of the main goals of the clinicians in the UC setting. Biomarkers of inflammation will attain growing importance in the clinic because we strive for more effective and cost-effective strategies to treat patients with IBD (28).

In our cohort, anti-TNF and VDZ were confirmed as good options for patients with UC in terms of efficacy: in our cohort, 62% of patients achieved clinical remission at 54 weeks, and even 46% displayed mucosal healing. IFX is the most studied drug in the UC setting, and its mucosal healing rate has been reported to range from 30% to 45% (26). ADA results in terms of mucosal healing are quite heterogeneous in the literature depending on the setting of patients: in clinical trials, it was approximately 25% at 1 year (8,29), whereas in real life, it ranged from 49% to 68% (30–32), reaching a surprising rate of 76% in case of moderate disease at baseline (33). At variance, GOL results in terms of mucosal healing are higher in clinical trials (54%–59%) (9,34) and quite heterogeneous in the 3 available real-life studies (19% at 6 months in an Italian cohort (35), 35% in 2 UK centers after induction (36), and 40% in Belgian patients at week 14 (37)). With regard to VDZ, mucosal healing ranges from 56%, as reported for maintenance treatment in the GEMINI I trial (11), to 69% in a US prospective study (38).

The main issue in the evaluation of mucosal healing is the time over which the clinician expects its achievement. In our opinion, an appropriate timing to evaluate the mucosal effect of a biologic drug in patients with UC should be at least 1 year, provided that patients display a clinical response to treatment: in the perspective of a treat-to-target strategy, we should allow enough time to drug therapy to express its full potential because if the target is not reached, the treatment should be optimized or changed. In this respect, because we have waited for 54 weeks to perform the follow-up endoscopy, we could obtain high rates of mucosal healing, even with subcutaneous drugs.

Loss of response was observed in 27% patients. These findings are in line with the current literature, where loss of response was reported to range from 23% to 46% 12 months after anti-TNF initiation (39), although a recent systematic review with meta-analysis showed a loss of response rate of 39.8 per 100 person-years of follow-up in patients treated with VDZ (15).

In our series, FC levels at week 8 showed a strong correlation with mucosal healing and clinical remission rates after 54 weeks of treatment. Various studies on patients with UC treated with anti-TNF, in particular IFX and ADA, have shown that a rapid decrease in FC levels over the initial weeks is positively associated with the rates of clinical remission and mucosal healing. Molander et al. (20) observed that FC ≤100 mg/kg after the induction of IFX or ADA therapy predicts clinical remission at 1 year: 139 mg/kg was identified as a cutoff to predict a risk of clinically active disease after 1 year, with a sensitivity of 72% and a specificity of 80%.

With regard to mucosal healing, a prospective multicenter study conducted in Belgium showed that FC <50 mg/kg at week 10 has a very good correlation with mucosal healing (evaluated at the same time point) in patients with UC treated with IFX (22). This finding was not completely unexpected, given the large number of studies that support a correlation between FC and the endoscopic activity of UC (18,19,40,41). FC has also been used to evaluate short-term endoscopic outcome in patients with UC, and a correlation with mucosal healing has been demonstrated (42,43). More interestingly, Guidi et al. (21) evaluated the predictive role of FC after the induction of IFX and ADA, showing that a value ≤168 mg/kg had a sensitivity of 83% and a specificity of 74% in predicting a sustained clinical response at 1 year, whereas a value ≤121 mg/kg had a sensitivity of 79% and a specificity of 57% in predicting mucosal healing. However, this study included both patients with UC and patients with Crohn's disease; the analysis was not stratified by disease, and the proportion of patients with UC was 30%. As a consequence, the data of patients with UC could not be singled out, and this was likely a major concern. By contrast, our cohort included only patients with UC, and this could lead to more convincing conclusions for this disease.

Data regarding FC in VDZ therapy are limited. A *post hoc* analysis of the GEMINI I trial (44) showed an FC decrease over the first 6 weeks of treatment that was more pronounced in patients under VDZ treatment than those treated with placebo. However, in this study, FC did not correlate with clinical and endoscopic outcomes evaluated after 6 weeks, even if a 90% reduction of FC levels had 89% specificity for mucosal healing. VDZ has been reported to induce disease remission slower than anti-TNF drugs (45). Thus, it is particularly important to wait a little longer to evaluate its endoscopic effectiveness. In this perspective, an early prediction of the therapeutic outcome with VDZ should lead to a better management of these patients. At present, the only parameter able to predict, with a good evidence, VDZ effectiveness, in terms of both endoscopic outcome (46) and treatment persistency (25), are serum drug levels. Serum cytokines have been proposed as well with this aim (47). However, the assessment of drug levels or serum cytokines for monitoring purposes is not available in all hospitals, and this would limit their use in routine clinical practice worldwide. However, our study supports a prospective role of FC evaluated over the first weeks of treatment, which is more suitable and easy to perform.

In our analysis, the ROC curve identified a value of 157.5 mg/kg with a sensitivity of 75% and a specificity of 89%. This finding is in line with previous studies. In 2005, our group showed that patients with FC <150 mg/kg had a lower risk of clinical relapses (48). Guardiola et al. (49) reported that a value >155 mg/kg correlated with histologic inflammation. Moreover, a recent study by Jha et al. (50) showed that a value of 158 mg/kg has a sensitivity of 90% and a specificity of 85% to predict mucosal healing in patients with UC.

Hassan et al. (51) evaluated FC and CRP in patients with UC after 12 weeks of treatment with IFX in conjunction with mucosal healing at the same time point and found a better correlation for FC, even though CRP also correlated significantly with mucosal healing. However, other data from the literature have shown that CRP is not as useful in UC, as it is in Crohn's disease, for the assessment of disease activity, with the exception of acute severe colitis (52,53). For instance, Arias et al. (54) found that baseline CRP ≤5 mg/L correlated with the therapeutic outcome to IFX therapy in UC, whereas a large Korean retrospective study showed that CRP ≥3 mg/dL was able to predict the therapeutic efficacy of this drug. With regard to VDZ therapy, despite various authors have investigated the role played by CRP, the results are not encouraging (55). In accordance with these conflicting findings, in our cohort, CRP at week 8 was not relevant in predicting mucosal healing at 54 weeks, even if it showed a possible role in predicting clinical remission at the same time point. Because mucosal healing is the target of treatment of UC (1), our study suggests the use of FC, instead of CRP, in therapeutic management to predict therapeutic effectiveness.

The main strength of the present study is the prospective design, which avoided several putative biases. Indeed, all patients performed all the assessments at the same time points (8 weeks after the induction for FC and CRP and 54 weeks after baseline for the endoscopic evaluation); moreover, all endoscopies were performed by 2 operators blinded to FC and CRP values. Of note, other important data are provided by our study because this is the first prospective real-life investigation where FC levels have been proven to have a role in the prediction of mucosal healing at 54 weeks in VDZ-treated patients. Moreover, the predictive role of FC is currently proposed only in a few studies conducted during IFX and ADA treatments, mainly with retrospective design, whereas the present prospective study has shown that its use is reliable for all anti-TNF drugs. Furthermore, the statistical strength of the correlation between FC at 8 weeks and mucosal healing, regardless of the mechanism of action of the drug, could allow to conclude that this fecal biomarker is useful as early predictor of response to all the biological therapies available in the UC setting, and therefore, its wider use should be encouraged.

The main limitation of this study is the lack of a multiple evaluation of FC: its levels can vary day-to-day, by the time of day, and even within the same bowel movement (56). Moreover, NSAID use or other inflammatory diseases could increase FC levels (23). Nevertheless, the data of this study should be considered a first step toward future studies with a greater number of patients and, above all, at least a double evaluation of FC at week 8.

In conclusion, we have documented an early predictive value of FC in patients treated with all the biological therapies currently available for the treatment of moderate/severe UC. This finding encourages the use of FC to obtain an early evaluation of treatment outcome. At present, this study suggests that patients with higher levels of FC at week 8 should be tightly monitored, regardless of clinical activity, likely anticipating colonoscopy, to optimize their treatment or even switching to another biological drug. Moreover, the present data pave the way to future investigations aimed at assessing more conclusively the value of FC levels in monitoring the therapeutic management of biological therapies in patients with UC.

## CONFLICTS OF INTEREST

**Guarantor of the article:** Lorenzo Bertani, MD.

**Specific author contributions:** L.B.: study concept and design, data collection, writing of the manuscript, and approving final version; C.B.: writing of the manuscript and approving the final version; M.G.M., L.C., E.A., G.T., G.B.S., F.Z., and F.C.: data collection and approving the final version; N.d.B. and M.B.: writing of the manuscript and approving the final version; R.M.: statistical analysis and approving the final version; S.M.: writing of the manuscript and approving the final version; and F.C.: study concept and design, writing of the manuscript, and approving the final version.

**Financial support:** This paper has not required funding in terms of grants, equipment, and drugs. No supportive foundations have funded this article.

**Potential competing interests:** F.C. received board membership honoraria from Takeda, Janssen, and Amgen and lecture fees from Abbvie, Takeda, Zambon, Ferring, Diasorin, Otsuka, and MSD; none of these honoraria had influence on this paper. All other authors have no potential conflict of interest in presenting this paper.

**Study Highlights**

**WHAT IS KNOWN**

✓ FC is widely used as a surrogate biomarker of endoscopic activity in UC.
✓ A reliable biomarker able to predict the therapeutic outcome to biological therapies is still missing.

**WHAT IS NEW HERE**

✓ An early assessment of FC is able to predict mucosal healing after 54 weeks of treatment.
✓ This is the first prospective study where FC showed a potential role as a prospective biomarker in predicting the therapeutic outcome in all biological therapies in UC.

**TRANSLATIONAL IMPACT**

✓ A simple ELISA test could be inserted in clinical practice to have an early prediction of treatment outcome and, possibly, guide treatment decisions.

## Supplementary Material

SUPPLEMENTARY MATERIAL
