# Supplementary figures and images for: Fecal Calprotectin Predicts Mucosal Healing in Patients With Ulcerative Colitis Treated With Biological Therapies: A Prospective Study
Source: Clin Transl Gastroenterol. 2020 May 18;11(5):e00174. doi: 10.14309/ctg.0000000000000174 (PMC7263645; doi:10.14309/ctg.0000000000000174)

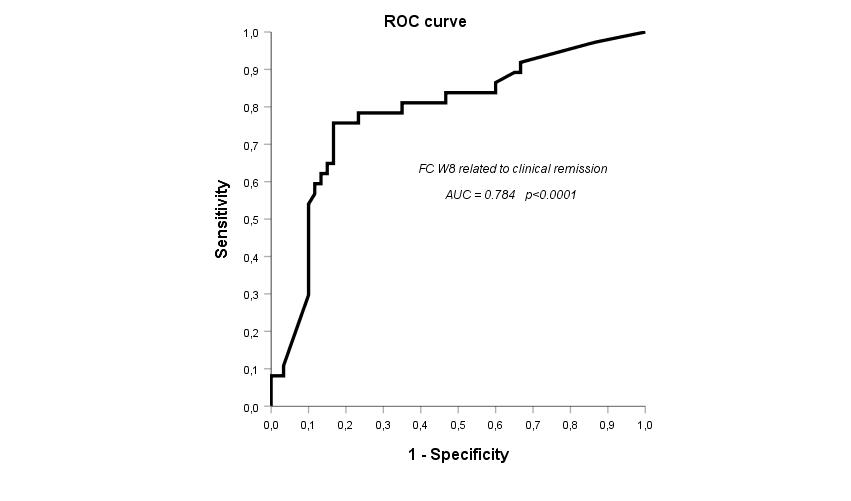

Supplement: SUPPLEMENTARY MATERIAL [file ct9-11-e00174-s001.tif]
